# A cluster randomised controlled trial of the Wellbeing in Secondary Education (WISE) Project – an intervention to improve the mental health support and training available to secondary school teachers: protocol for an integrated process evaluation

**DOI:** 10.1186/s13063-018-2617-4

**Published:** 2018-05-04

**Authors:** Rhiannon Evans, Rowan Brockman, Jillian Grey, Sarah Bell, Sarah Harding, David Gunnell, Rona Campbell, Simon Murphy, Tamsin Ford, William Hollingworth, Kate Tilling, Richard Morris, Bryar Kadir, Ricardo Araya, Judi Kidger

**Affiliations:** 1 DECIPHer, School of Social Sciences, Cardiff University, 1-3 Museum Place, Cardiff, CF10 3BD UK; 2 Bristol Medical School, Population Health Sciences, University of Bristol, Canynge Hall, 39 Whatley Road, Bristol, BS8 2PS UK; 3 University of Exeter Medical School, University of Exeter, St Luke’s Campus, Heavitree Road, Exeter, EX1 2LU UK; 4 Centre for Global Mental Health and Primary Care Research, Institute of Psychiatry, Psychology & Neuroscience, King’s College London , David Goldberg Centre, De Crespigny Park, London, SE5 8AF UK

**Keywords:** Mental health, Wellbeing, Schools, Children, Adolescents, Teachers, Process evaluation, Cluster Randomised Controlled Trial

## Abstract

**Background:**

Secondary school teachers have low levels of wellbeing and high levels of depression compared with the general population. Teachers are in a key position to support students, but poor mental health may be a barrier to doing so effectively. The Wellbeing in Secondary Education (WISE) project is a cluster randomised controlled trial (RCT) of an intervention to improve the mental health support and training available to secondary school teachers through delivery of the training package Mental Health First Aid and a staff peer support service. We will conduct a process evaluation as part of the WISE trial to support the interpretation of trial outcomes and refine intervention theory. The domains assessed will be: the extent to which the hypothesised mechanisms of change are activated; system level influences on these mechanisms; programme differentiation and usual practice; intervention implementation, including any adaptations; intervention acceptability; and intervention sustainability.

**Methods:**

Research questions will be addressed via quantitative and qualitative methods. All study schools (*n* = 25) will provide process evaluation data, with more detailed focus group, interview and observation data being collected from a subsample of case study schools (4 intervention and 4 control). Mechanisms of change, as outlined in a logic model, will be measured via teacher and student surveys and focus groups. School context will be explored via audits of school practice that relate to mental health and wellbeing, combined with stakeholder interviews and focus groups. Implementation of the training and peer support service will be assessed via training observations, training participant evaluation forms, focus groups with participants, interviews with trainers and peer support service users, and peer supporter logs recording help provided. Acceptability and sustainability will be examined via interviews with funders, head teachers, trainers and peer support services users, and focus groups with training participants.

**Discussion:**

The process evaluation embedded within the WISE cluster RCT will illuminate how and why the intervention was effective, ineffective or conferred iatrogenic effects. It will contribute to the refinement of the theory underpinning the intervention, and will help to inform any future implementation.

**Trial registration:**

International Standard Randomised Controlled Trial Number: ISRCTN95909211 registered on 24 March 2016.

## Background

Teaching professionals are at an increased risk of common mental health disorders compared with other occupations [1, 2]. Findings from the Wellbeing in Secondary Education (WISE) pilot study found that, among a sample of 555 secondary school teachers, scores on the Warwick Edinburgh Mental Wellbeing Scale were approximately four points below the average of the general working population [3]. Additionally, 19.4% reported experiencing moderate to severe levels of depression on the Patient Health Questionnaire (PHQ-9), compared with a general population prevalence of 8–10% [4, 5]. Poor mental health amongst teachers is associated with adverse work-related outcomes such as absenteeism, presenteeism and health-related workplace retirement [6–8].

Failure to address teachers’ mental health and wellbeing can detrimentally influence student health. Poor teacher-student relationships in secondary school predict student psychiatric disorders and later exclusion [9]. Meanwhile, positive relationships are associated with lower levels of student depression and increased educational achievement [10, 11]. Teachers are also the professionals most likely to have routine contact with students in regard to their mental health [12]. However, poor wellbeing reduces teachers’ belief in their ability to support students [13], with this problem being compounded by a lack of training in how to effectively do so [14]. In turn, this threatens teachers’ own mental health, as they recognise their unfulfilled potential to help [15].

To date, there have been limited interventions aiming to support teacher mental health, with most focusing on teachers’ competencies in supporting students. Two large scale randomised controlled trials (RCTs) have evaluated the effect of providing teachers with mental health training within secondary school settings. A cluster RCT evaluated the impact of delivering Youth Mental Health First Aid training to school staff [16]. Standard Mental Health First Aid (MHFA) training aims to train lay people to recognise the signs and symptoms of common mental health problems, while the youth version of the programme is targeted at those who work with individuals aged 8–18 years. The study found positive changes in staff mental health knowledge, attitudes and confidence in helping young people. However, no changes were reported in actual helping behaviours or student mental health. More recently, the Saving and Empowering Lives in Europe (SEYLE) project, a three-arm RCT across 10 European countries, compared the effectiveness of training teachers to recognise and support students at risk of suicide, raising student awareness about mental health and suicide, and screening by professionals [17]. The teacher training element did not demonstrate a large effect, and the authors suggested that poor teacher wellbeing may have reduced their ability to support students [13]. Neither of these secondary school-based interventions included a component to improve the mental health of teachers themselves; therefore, there is an evident need to develop interventions that address this outcome.

We conducted a pilot RCT of an intervention to improve the mental health and wellbeing of teachers and improve their skills in supporting students across six secondary schools in England [18]. From the study results, we deemed it would be feasible and justifiable to conduct a full-scale cluster RCT, with an embedded process and economic evaluation. The protocol for the cluster RCT has been reported elsewhere [19]. This paper outlines the protocol for the embedded process evaluation.

### The Wellbeing in Secondary Education (WISE) intervention

The WISE intervention’s primary aim is to improve the mental health and wellbeing of teachers through provision of a peer support service and training in supporting students. The intervention’s theory of change is informed by social support theory. Social support offers problem-focused coping strategies and emotion-focused supportive strategies, both of which can have a positive impact on physical and mental health [20]. Based on findings from the pilot study [18], we hypothesise that peer supporters will provide both emotion-focused support, for example, by listening non-judgementally, and problem-focused support, for example, by offering practical suggestions for solutions to work-based difficulties where appropriate. Perceived availability of social support may be even more important to mental health than actual support [20], and therefore the existence of a peer-delivered support service is theorised to have a positive impact on teacher wellbeing, regardless of actual service utilisation. The theory of change is further informed by an ecological view of school connectedness, which considers the quality of social bonds and interactions within a school to be a characteristic of the whole school environment or culture [21]. Improvement to teachers’ own mental health and wellbeing via supportive relationships with peers should lead to more positive teacher–student relationships [22], which is associated with improved student mental health [9]. Thus, all teachers and students within an intervention school may benefit, regardless of whether they themselves directly engage with the intervention.

The WISE intervention involves three components, namely a staff peer support service, teacher training in MHFA, and a teacher mental health awareness raising session.

#### Staff peer support service

School staff nominated by their colleagues (8% of staff body, with a maximum of 16) will attend the 2-day standard MHFA training. This proportion of the population has been used in successful peer influence interventions [23] and we hypothesise that training this number of peers will mean that all staff members have access to someone whom they feel comfortable approaching for support. The one exclusion criterion for nomination will be membership of the Senior Leadership Team, as pilot findings indicated that staff might feel uncomfortable using a support service that includes senior leaders. The 8% of staff with the most nominations, whilst ensuring a mix of gender and teaching/non-teaching roles, will be invited to attend the standard MHFA training. The training educates participants to spot the signs and symptoms of mental health problems, and to know how to respond to an individual in distress or need of support. It focuses on the ALGEE model, namely to Assess for risk of suicide or other harm, Listen non-judgementally, Give reassurance and information, Encourage appropriate professional help-seeking, and Encourage self-help and other support strategies [24]. Guidance developed by the study team will be presented during the training on how to set up and run a peer support service, although each peer support service will have the autonomy to develop their own advertising and service implementation strategies to ensure best fit for the context of their particular school. Following completion of the training course, staff will establish a confidential peer support service for colleagues offering informal support and signposting to other services where appropriate.

#### Teacher training in MHFA for schools and colleges

Teaching staff (8% of teaching staff, with a maximum of 16) with pastoral roles such as personal tutor or head of year will attend the 1-day MHFA for Schools and Colleges training. The course is based on the Youth MHFA training, and also covers ALGEE, but focuses particularly on the school setting. Trained teachers will continue with their usual teaching and pastoral roles within school, but will apply the MHFA for Schools and Colleges learning in their day-to-day interactions with students, responding to signs and symptoms of distress and providing initial help and support to individuals they identify to be at risk of mental health difficulties.

#### Teacher mental health awareness raising session

All teaching staff will receive a 1-hour awareness raising session, which will highlight the importance of mental health in schools, offer advice on how to support the mental health of self and others, and introduce the peer support service. The session has been designed through collaboration between the study team and MHFA trainers.

### Implementation strategy

Different models for implementation will be used in England and Wales, due to differing funding structures and a desire to make use of existing resources and infrastructure to maximise intervention sustainability, if effective. In Wales, training will be delivered to schools by Healthy School Coordinators, who will first complete a 6-day bespoke version of the MHFA instructor course. Healthy School Coordinators are employed by Local Authorities or Public Health Wales and are responsible for monitoring and accrediting schools participating in the Welsh Network of Healthy Schools Scheme. Seven Healthy School Coordinators will be trained to ensure sufficient capacity for intervention delivery. In England, training will be delivered by accredited MHFA trainers.

We will aim to deliver the three training components to each school within one academic term (September to December 2016). The order in which training components are delivered is not pre-specified due to the need to fit with schools’ pre-planned training dates. Each training component includes standardised slides. However, for the MHFA training, trainers can choose to utilise supplementary activities and modes of learning (e.g. group discussion, videos, exercises) from a suite of options. Peer supporters will meet within 3–4 weeks of the standard MHFA training course to establish the peer support service.

### Process evaluation aims

Process evaluations embedded within RCTs support the interpretation of trial outcomes and the refinement of intervention theory [25]. Recent Medical Research Guidance on process evaluation offers the most comprehensive and developed instruction on their conduct within effectiveness trials [25]. While recognising the need for a flexible evaluation model, the guidance specifies key process evaluation components, which reflect the underpinning realist and complex  systems principles [26–28].

#### Mechanisms of change

**Fig. 1:**
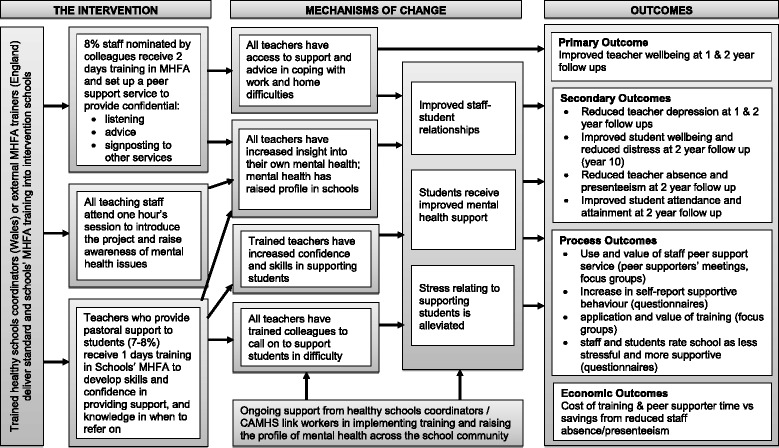
Wellbeing in Secondary Education (WISE) logic model

We will examine if the intervention’s hypothesised mechanisms of change are activated, and the extent to which these mechanisms are modified though their interaction with contextual characteristics. Postulated mechanisms, refined through the feasibility and pilot phase of intervention evaluation, are depicted in the logic model (Fig. 1). In adherence with recommendations for the generation and testing of dark logic models, in which potential adverse outcomes are considered [29], data will examine iatrogenic causal pathways.

#### Differentiation and usual practice

We will monitor usual practice across study schools to assess programme differentiation in intervention schools and ascertain if contamination has occurred in control schools.

#### Intervention implementation

We will conduct a multi-component assessment of implementation, comprising (1) the reach of the WISE intervention training course and the peer support service; (2) completion of the WISE intervention training course (dosage); (3) fidelity to the planned intervention during delivery of the WISE intervention training course and peer support service, defined as adherence to the intervention’s core processes. This includes coverage of core MHFA materials and learning objectives during training delivery, the extent to which the peer support service is established and run in accordance with the plan developed in the pilot, and the extent to which peer supporters utilise their MHFA learning during delivery; (4) quality of delivery, including the quality of training provided by trainers (which is a measurement of knowledge, presentation skills and facilitation skills) and the quality of support provided by the peer support service; and (5) barriers and facilitators to implementation of the peer support service in the school context.

#### Acceptability

We will explore intervention acceptability by assessing participants’ perceptions and experiences of the training and peer support service, and how this differs across school contexts and across the course of the delivery (e.g. whether acceptability to peer supporters changes through the process of delivering the peer support service) [25, 30].

#### Sustainability

We will consider the extent to which the intervention is sustainable by assessing its scope to become part of usual practice outside of a trial setting [31], and examining the contextual factors that may determine decision-making around continuance. This will include consideration of the continued presence of peer supporters, and the feasibility of recruiting and training new staff members if existing supporters have left the school.

Table 1 presents the research questions addressed by the process evaluation, and how they map onto the five domains being examined.

**Table 1:** Process evaluation domains and research questions

Process evaluation domain	Research question
Mechanisms of change	RQ1: Are the intervention’s mechanisms of change operationalised as hypothesised? RQ2: How is the operationalisation of the mechanisms of change influenced by contextual factors? RQ3: Does the interaction of the mechanisms of change with contextual factors give rise to unintended effects?
Programme differentiation and usual practice	RQ4: Is the Wellbeing in Secondary Education (WISE) intervention differentiable from ‘usual practice’ and does this differentiation change during the study? RQ5: Is there contamination of usual practice in control schools by receipt of the WISE intervention or similar approaches?
Implementation (WISE training components)	RQ6: What is the reach of the WISE training components (e.g. 8% of staff attending Standard Mental Health First Aid)? RQ7: How many targeted staff complete the WISE intervention training? RQ8: Are the WISE training components delivered with fidelity and what is the nature of any adaptions undertaken? RQ9: Are there differences in the delivery of the WISE training components between England and Wales, and what gives rise to any differences? RQ10: How well are the WISE training components delivered?
Implementation (peer support service)	RQ11: What proportion of teachers receive support from the peer support service? RQ12: Is the peer support service delivered with fidelity and what is the nature of any adaptions undertaken? RQ13: What are the barriers and facilitators to the implementation of the peer support service?
Acceptability	RQ14: Is the WISE intervention acceptable to funding organisations, intervention trainers, head-teachers, teachers and students?
Sustainability	RQ15: How likely is the WISE intervention to be sustainable and what factors might ensure sustainability?

## Methods

### Study design and sample

The study is a cluster RCT with an embedded process and economic evaluation [19]. The process evaluation will adopt a mixed methods approach and utilise both quantitative and qualitative data sources. We will collect process data alongside the outcome data. Data collection commenced with baseline measures in May 2016 and will be completed in July 2018.

The trial sample size and sampling strategy at the school level is reported in the main study protocol [19]. The study sample comprises secondary schools across the two study sites of England and Wales. Twenty-four schools were required to ensure statistical power, but 25 were recruited to mitigate against the risk of drop-out. Schools have been stratified by site (England or Wales), administrative region (educational consortia in Wales and local authority in England) and proportion of students eligible for free school meals (FSM) (high, medium or low compared to the national average), which is a proxy measure of socioeconomic status. Twelve schools have been randomly allocated to the intervention arm and 13 to the control arm.

All 25 study schools will provide process evaluation data. Four intervention and four control schools have been purposively sampled to serve as ‘case study schools’ and more extensive process evaluation will be undertaken in these cases. To sample the case study schools, the 25 schools were stratified by trial arm allocation (intervention or control), site (England or Wales), administrative region (educational consortia or local authority) and proportion of students eligible for FSM (high/low compared to the national average). One intervention school within each stratum was then purposively sampled to achieve variation in school size and assessment ranking by the educational inspectorate. One control school was selected within each stratum to match the intervention cases as closely as possible. Table 2 presents the final sample of case study schools.

**Table 2:** Sample for case study schools

School	Trial status	Site	Administrative region	Free school meal eligibility	School size	Inspectorate assessment
School 1	Intervention	England	1	Low	Large	Good
School 2	Intervention	England	2	High	Small	Inadequate
School 3	Intervention	Wales	3	High	Small	Adequate
School 4	Intervention	Wales	4	Low	Large	Good with outstanding features
School 5	Control	England	1	Low	Large	Requires improvement
School 6	Control	England	2	High	Small	Good
School 7	Control	Wales	3	High	Small	Adequate
School 8	Control	Wales	4	Low	Large	Good

Within-school sampling of individuals to participate in data collections will be random where response bias may be a risk (e.g. focus groups with non-trained teachers and with students), but otherwise will be purposive to ensure recruitment of a diverse range of views. Data generation will be systematically reviewed in order to check for theoretical saturation. If saturation has not been reached, and resources permit, further data collection will be undertaken.

#### Data sources

The process evaluation data to be provided by study schools, and how they relate to the research questions, is summarised in Table 3.

**Table 3:** Summary of process evaluation research questions and data sources

Research domain	Research question	Data source	Informant	Procedure of data collection	Time of data collection	Analysis
Mechanisms of change	RQ1: Operationalisation of intervention mechanisms RQ2: Contextual factors RQ3: Unintended effects	A. Teacher questionnaires; student questionnaires	Teachers (*n* ~ 1440) Students (*n* ~ 3600)	Self-assessment; survey questions regarding school-related stress and satisfaction, support and quality of relationships; perceptions of school caring about wellbeing	Baseline; 12 mth follow-up (teachers); 24 mth follow-up	Logistic regression examining impact on outcomes (part of main analysis)
		B. Audit of school polices	School Contact (*n* = 25)	Self-assessment; paper audit	Baseline; 24 mths follow-up	Tables of activities/policies; narrative description
		D. Observation of intervention training courses	WISE trainers (*n* = 10); training attendees (*n* = TBC; intervention case study schools *n* = 4)	Independent assessment of intervention training course by study team (*n* = 2); completion observation schedules	During intervention training course	Summaries of scores (means/medians); narrative description; inter-rater reliability
		G. Peer supporter log and feedback	Peer supporters (*n* = TBC, intervention schools *n* = 12)	Self-assessment; logs; feedback session hosted by study team	Termly following intervention training course; 2 × feedback sessions	Tables and summaries of quantitative data; thematic analysis
		I. WISE trainer interview	WISE trainer (*n* = 6)	Interview led by study team	Within 6 mths post-training	Thematic analysis
		J. Head-teacher interview	Head teachers (*n* = 25)	Interview led by study team	From 6 mths post-training	Thematic analysis
		K. Peer supporter and schools MHFA attendee focus groups	Training course attendees (*n* = 4–8 staff, intervention case study schools *n* = 4)	Focus group led by study team	6 mths post-training; 18 mths post-training	Thematic analysis
		L. Recipient of peer support service interview	Peer support recipients (*n* = 5 staff, intervention case study schools *n* = 4)	Interview led by study team	12 mths post-training	Thematic analysis
		M. Teacher focus group	Teachers (random sample 4–8 staff, intervention and control case study schools *n* = 8)	Focus group led by study team	12 mths post-training	Thematic analysis
		N. Year 10 focus group	Students (*n* = 6–8 students, intervention and control case study schools *n* = 8)	Focus group led by study team	12 mths post-training	Thematic analysis
Programme differentiation and usual practice	RQ4: Differentiation RQ5: Contamination	A. Teacher questionnaires; student questionnaires	Teachers (*n* ~ 1440) Students (*n* ~ 3600)	Self-assessment; survey questions regarding training and support received through school	Baseline; 12-mth follow-up (teachers); 24-mth follow-up	Tabulate responses
		B. Audit of school polices	School Contact (*n* = 25)	Self-assessment; paper audit	Baseline; 24-mth follow-up	Tables of activities/policies; narrative description
		J. Head-teacher interview	Head-teachers (*n* = 25)	Interview led by study team	From 6 mths post-training	Thematic analysis
		L. Recipient of peer support service interview	Peer support recipients (*n* = 5 staff, intervention case study schools *n* = 4)	Interview led by study team	12 mths post-trainings	Thematic analysis
		M. Teacher focus group	Teachers (random sample 4–8 staff, intervention and control case study schools *n* = 8)	Focus group led by study team	12 mths post-trainings	Thematic analysis
		N. Year 10 focus group	Students (*n* = 6–8 students, intervention and control case study schools *n* = 8)	Focus group led by study team	12 mths post-trainings	Thematic analysis;
Implementation (Training)	RQ6: Reach RQ7: Completion RQ8/RQ9: Fidelity RQ10: Quality	C. Attendance records	Trainers (*n* = 10)	Course registers	During intervention training course	Tabulate attendees
		D. Observation of intervention training courses	WISE trainers (*n* = 10), training attendees (*n* = TBC; intervention case study schools *n* = 4)	Independent assessment of intervention training course by study team (*n* = 2); observation schedules	During intervention training course	Summaries of scores (means/medians); narrative description; inter-rater reliability
		E. Fidelity checklist and training materials used	WISE trainers (*n* = 10), training attendees (*n* = TBC; intervention schools *n* = 12)	Self-assessment; checklists and materials log	During intervention training course	Summaries of scores (means/medians); tabulate materials used
		F. Training evaluation form	Training attendees (*n* = TBC; intervention schools *n* = 12)	Self-assessment; evaluation forms	Following intervention training	Summaries of scores (means/medians); paired *t*-tests
		J. Head-teacher interview	Head-teachers (*n* = 25)	Interview led by study team	From 6 mths post-training	Thematic analysis
		I. WISE trainer interview	WISE trainer (*n* = 6)	Interview led by study team	I. WISE trainer interview	Thematic analysis
Implementation (Peer support service)	RQ11: Reach RQ12: Fidelity RQ13: Barriers and facilitators	A. Teacher questionnaires; student questionnaires	Teachers (*n* ~ 1440) Students (*n* ~ 3600)	Self-assessment; survey questions regarding use of peer support service	Baseline; 12-mth follow-up (teachers); 24-mth follow-up	Tabulate responses
		G. Peer supporter log and feedback	Peer supporters (*n* = TBC; intervention schools *n* = 12)	Self-assessment; logs; feedback session hosted by study team	Termly following intervention training course; 2 × feedback sessions	Tables and summaries of quantitative data; thematic analysis
		J. Head-teacher interview	Head teachers (*n* = 25)	Interview led by study team	From 6 mths post-training	Thematic analysis
		K. Peer supporter and schools MHFA attendee focus group	Training course attendees (*n* = 4–8 staff; intervention case study schools *n* = 4)	Focus group led by study team	6 mths post-training; 18 mths post-training	Thematic analysis
		M. Teacher focus group	Teachers (random sample 4–8 staff, intervention and control case study schools *n* = 8; intervention case study schools *n* = 4)	Focus group led by study team	12 mths post-training	Thematic analysis
Acceptability	RQ14: Acceptability	G. Peer supporter log and feedback	Peer supporters (*n* = TBC, intervention schools *n* = 12)	Self-assessment; logs; feedback session hosted by study team	Termly following intervention training course; 2 × feedback sessions	Tables and summaries of quantitative data; thematic analysis
		H. Funding organisation interview	Funding organisation representative (*n* = 3)	Interview led by study team	From 6 mths post-training	Thematic analysis
		I. WISE trainer interview	WISE trainer (*n* = 6)	Interview led by study team	Within 6 mths post-training	Thematic analysis
		J. Head teacher interview	Head-teachers (*n* = 25)	Interview led by study team	From 6 mths post-training	Thematic analysis
		K. Peer supporter and schools MHFA attendee focus group	Training course attendees (*n* = 4–8 staff; intervention case study schools *n* = 4)	Focus group led by study team	6 mths post-training; 18 mths post-training	Thematic analysis
		L. Recipient of peer support service interview	Peer support recipients (*n* = 5 staff, intervention case study schools *n* = 4)	Interview led by study team	12 mths post-training	Thematic analysis
		M. Teacher focus group	Teachers (random sample 4–8 staff; intervention and control case study schools *n* = 8)	Focus group led by study team	12 mths post-training	Thematic analysis
		N. Year 10 focus group	Students (*n* = 6–8 students; intervention and control case study schools *n* = 8)	Focus group led by study team	12 mths post-trainings	Thematic analysis
Sustainability	RQ15: Sustainability	H. Funding organisation interview	Funding organisation representative (*n* = 3)	Interview led by study team	From 6 mths post-training	Thematic analysis
		I. WISE trainer interview	WISE trainer (*n* = 6)	Interview led by study team	Within 6 mths post-training	Thematic analysis
		J. Head-teacher interview	Head-teachers (*n* = 25)	Interview led by study team	From 6 mths post-training	Thematic analysis
		K. Peer supporter and Schools MHFA attendee focus group	Training course attendees (*n* = 4–8 staff; intervention case study schools *n* = 4)	Focus group led by study team	6 mths post-training; 18 mths post-training	Thematic analysis
		L. Recipient of peer support service interview	Peer support recipients (*n* = 5 staff; intervention case study schools *n* = 4)	Interview led by study team	12 mths post-training	Thematic analysis
		M. Teacher focus group	Teachers (random sample 4–8 staff; intervention and control case study schools *n* = 8)	Focus group led by study team	12 mths post-training	Thematic analysis

*MHFA* Mental Health First Aid, *TBC* to be confirmed, *WISE* Wellbeing in Secondary Education

##### Teacher and student questionnaire

All 25 schools will complete teacher and student questionnaires. Teacher questionnaires will be completed at baseline and at 12- and 24-month follow-up. Student questionnaires will be completed by Year 8 students at baseline and at 24-month follow-up (when in Year 10). Data will test the postulated theory of change underpinning the intervention through measurement of stress and satisfaction at school, support and quality of relationships, and perceptions of whether the school cares about teacher and student wellbeing. Follow-up questionnaires will also examine intervention reach (numbers who have completed MHFA training, and who have received support from the peer supporters) and contamination.

##### Audit of school policies and interventions

The 25 study schools will be audited at baseline and at 24-month follow-up. We will ask the main contact teacher for each school to collect evidence of existing policies and interventions being undertaken in relation to the mental health and wellbeing of teachers and students. The baseline audit will permit comparison of pre-existing activities in intervention and control schools to explore possible baseline imbalances and generate hypotheses as to how the context may impact upon the operationalisation of the intervention’s theory of change. The follow-up audit will record relevant policies or interventions introduced during the study period. It will explore programme differentiation from usual practice in intervention schools and assess contamination in the control schools by identifying the extent to which any new activities resemble components of the WISE intervention.

##### Attendance records for the WISE intervention training

Attendance records for the training courses will be generated. This will assess reach (numbers completing the training). The intervention stipulates that a minimum of 8% of school staff attend the standard MHFA training course and a minimum of 8% of teachers attend the Schools MHFA training course. The proportion of staff attending the 1-hour awareness raising session will also be noted.

##### Observations of the WISE intervention training

In the four intervention case study schools, observations of the 1-hour awareness raising session, the standard MHFA training course, and the MHFA for Schools and Colleges training course, will be conducted. Two members of the research team will independently observe all sessions. Standardised observation schedules will be completed to quantitatively assess coverage of materials, quality of delivery and participant engagement. Coverage will be assessed by a binary score and the remaining items by a 5-point Likert scale. Observers will qualitatively document any information relevant for understanding the quantitative assessment, and other issues of importance not covered by the quantitative scale, including course adaptations and general contextual observations. Observation data will inform the topic guides utilised during focus groups and interviews with intervention trainers, peer supporters and attendees at the MHFA for Schools and Colleges training course.

##### Post-training course fidelity checklist and record of materials

Following the completion of the standard MHFA and MHFA for Schools and Colleges training course, participants across the 12 intervention schools will be asked to complete a checklist recording the activities and modes of learning utilised. In addition, each trainer will complete a record of materials used, in which they list any resources, activities or examples that were additional to the standardised slides. These data will be used to assess fidelity and compare variations in the intervention training delivery across schools.

##### Training evaluation forms

In the 12 intervention schools, attendees at the 1-hour awareness raising session, standard MHFA, and MHFA for Schools and Colleges training courses will complete evaluation forms. The MHFA courses issue standardised evaluation forms administered on completion of all MHFA training packages. Data will be extracted from these forms for the purposes of the study. The evaluation forms record any self-reported increase in knowledge and confidence in supporting others, and views on course quality. A study-specific evaluation form will be developed for the 1-hour awareness raising session to also assess coverage of learning materials and quality of delivery.

##### Peer supporter logs and feedback sessions

In the 12 intervention schools, peer supporters will be asked to complete a termly electronic log documenting delivery of support to colleagues during the previous two working weeks. There will be some variation in the time-point of log completion during the academic term in order to mitigate against the risk of seasonal bias (e.g. stress associated with end of term examinations). The log will assess reach, the broad demographic and professional characteristics of the staff members supported, the type of problem addressed (e.g. work- or personal-related), and the outcome of the interaction.

A subgroup of peer supporters will be invited to attend a meeting with the study team at approximately 6 and 18 months post training. These sessions will monitor peer support service processes to aid assessment of fidelity, consider acceptability and explore potential adverse events.

##### Interviews with funding organisation representatives

Semi-structured interviews will be conducted with a representative from each funding organisation that has contributed to intervention costs (*n* = 3). These are Bristol City Council, Public Health England and Public Health Wales. Interviews will assess intervention acceptability, fit with existing organisational priorities and the feasibility of sustained resource allocation to the WISE intervention if found to be effective.

##### Interviews with trainers

Semi-structured interviews will be conducted with a subgroup of the trainers. Participants will include the three MHFA trainers in England and three of the seven Healthy Schools Coordinators in Wales. The Coordinators will be purposively sampled to ensure that a representative from each of the six intervention schools is interviewed (coordinators delivered the intervention in pairs). Data will explore mechanisms underpinning the learning processes, preparedness to deliver the three intervention components, experiences of delivery, including barriers and facilitators, fidelity and motivations for any adaptations undertaken, perceived acceptability, and willingness to contribute to future delivery if the intervention is effective.

##### Interviews with head-teachers

Semi-structured interviews will be conducted with the head-teachers of the 25 study schools. Data will explore the school context, including the perceived role of schools in addressing the mental health and wellbeing of staff and students, the acceptability of usual practice, and the barriers and facilitators associated with the delivery of relevant policies and interventions. In the 12 intervention schools, interviews will also explore contextual influences on the intervention’s mechanisms of change, implementation, acceptability and sustainability.

##### Focus groups with peer supporters and recipients of MHFA for Schools and Colleges training

In the four intervention case study schools, focus groups will be conducted with school staff who have attended the standard MHFA training and taken on the role of peer supporter and with teachers who have attended the MHFA for Schools and Colleges training. Two waves of focus groups will be undertaken, the first within 6 months of training delivery, and the second within 18 months of training delivery. Focus groups will explore the acceptability of MHFA training and attendees’ preparedness to become peer supporters or to support students, the acceptability of delivering support, barriers and facilitators to implementation of the training knowledge and peer support service, fidelity to the intended model and reasons for adaptations undertaken, and the potential sustainability of the intervention. Each group will comprise four to eight participants and selection will be purposive to ensure variation in gender and role. Where individuals do not wish to express their views within a group setting, one-to-one interviews will be offered as an alternative.

##### Interviews with peer support service users

In the four intervention case study schools, semi-structured interviews will be conducted with teachers who have utilised the peer support service once the WISE intervention has been delivered for approximately one academic year. Approximately five teachers will be interviewed at each school, depending on the point at which theoretical saturation is reached and no new themes emerge from the data. Participants will be recruited through the peer supporters where possible, or via an email to all staff and/or an announcement at staff meetings. Topic guides will cover the perceived outcomes of utilising the peer support services and the mechanisms through which these outcomes have been realised, the acceptability of the support offered, including comparison to ‘usual practice’ and the intention to utilise the support service again, and perceived barriers and facilitators to uptake.

##### Focus groups with teachers not in receipt of the MHFA training

In the four intervention case study schools and the four control case study schools, focus groups will be conducted with teachers who did not receive any MHFA training once the WISE intervention has been delivered for approximately one academic year. Each focus group will comprise four to eight teachers who have been randomly sampled. In the intervention schools, focus groups will explore views of the peer support service, including awareness, acceptability and its comparison to ‘usual care’, perceived barriers and facilitators to uptake, and potential service sustainability. In the control schools, focus groups will explore evidence of contamination, perceptions of ‘usual practice’ and views on a hypothetical peer support service. Where teachers do not wish to express their views within a group setting, one-to-one interviews will be offered as an alternative.

##### Focus groups with Year 10 students

In the four intervention case study schools and the four control case study schools, focus groups will be conducted with Year 10 students, who would have completed the baseline questionnaire in Year 8. Focus groups will be conducted when the WISE intervention has been delivered for approximately one academic year. Each focus group will comprise six to eight students. A random sample of six students (mixed gender) will be identified, and students will be invited to nominate a friend within the same year to also attend. We will aim to recruit a higher number of students than required, to allow for drop out on the day. In the intervention schools, students are unlikely to be aware of the intervention, as the hypothesised impact on them will be indirect, via more supportive teachers, therefore topic guides for both intervention and control schools will be the same. Focus groups will explore participants’ views of teacher–student relationships and provision of mental health and wellbeing support in their schools. Where students do not wish to express their views within a group setting, one-to-one or paired friendship interviews will be offered as an alternative.

#### Analysis

Process evaluation data will be analysed independently of the trial outcome data. The datasets will be integrated following the completion of outcome data analysis so that the process evaluation can inform the interpretation of these data. At the point of analysis, members of the research team undertaking process data analysis will be blinded to the outcome dataset and the trial statisticians will be blinded to the process evaluation dataset.

### Qualitative analysis

Qualitative data will be audio recorded and transcribed verbatim. The qualitative data analysis software package NVivo 11 will be used to support analysis and data management. Thematic analysis will be conducted [32, 33]. Two researchers will index a subset of data to construct a coding framework, with one framework constructed for each dataset (peer supporters, students, etc.). A priori codes that map onto the process evaluation domains will be included in the coding framework, along with novel codes that emerge from the data. The remaining corpus of data will then be analysed according to the coding framework, with refinements being made to the frameworks as new themes emerge. A second member of the research team will independently double code 10% of the data. Once all data are coded, candidate themes will be identified for each dataset. Themes from across all datasets will then be compared and refined to agree a set of final study-level themes. Integration and triangulation of data will adopt a complementary approach, whereby all participant narratives are equally privileged in the generation of new theoretical and empirical insights [34]. Anonymised data will be presented in the form of quotes to illustrate each theme.

### Quantitative analysis

Observational data reporting on implementation will be summarised using descriptive statistics, which will enable examination of between and within school variation. The possibility of summarising all data to construct an implementation score, to identify high versus low implementing schools, will be explored. We do not make an a priori specification of a high/low implementation threshold as it is both relative and study specific [35]. Implementation data will be combined with outcome data to inform the interpretation of variation in school-level outcomes, specifically where there may be outliers.

As part of the main trial analysis, logistic regression models will be undertaken to compare binary measures from the teacher and student questionnaires between arms at follow-up. The binary measures are teachers’ stress and satisfaction at work, support provided/received by teachers and students at school, perceived attitude of schools to staff and student wellbeing, and perceived quality of relationships in schools. Adjustment will be made for baseline scores, school-level FSM and administrative region. Analyses will examine whether these variables moderate the effect of the intervention on teacher wellbeing [19]. However, this analysis will be exploratory as power will be low, and there is potential for unmeasured confounding.

#### Ethics and consent

Ethical approval for the study has been granted by the University of Bristol’s Faculty of Health Sciences Research Ethics Committee (FREC reference number: 28522). The consent procedure for schools to join the study and for participants to complete a questionnaire is reported in the main protocol paper [19]. For the interviews and focus groups conducted as part of the process evaluation, all those invited to take part will receive a participant information leaflet at least 2 weeks prior to data collection. Written consent will be obtained by the study team prior to any data collection taking place. Parents of students taking part in focus groups will also be supplied with an information sheet, and will be asked to give consent for their child to take part. Consent will either be written or verbally obtained by the school contact, which they will formally record in writing. For those invited to take part in the study as peer supporters, written information will be provided at least 2 weeks prior to the training delivery, and written consent will be obtained before the training is attended.

## Discussion

This paper presents a detailed protocol for the process evaluation of a complex school-based intervention intended to improve the mental health and wellbeing of teachers. We will address five key evaluation domains, namely (1) mechanisms of change and their interaction with system-level influences; (2) programme differentiation and usual practice; (3) implementation and adaptation; (4) acceptability; and (5) sustainability. Process data will support the interpretation of the outcome data from the RCT [20], through the illumination of how and why the intervention was effective, ineffective or conferred iatrogenic effects. It will further contribute to the development and refinement of the intervention theory, and support further understanding of the mechanisms through which peer support models may impact upon both teacher and student mental health and wellbeing. Data will feed into the dissemination plan and may support the longer-term implementation of the intervention, depending on effectiveness. The evaluation will also offer additional insight into the complexity of the school system, and how the system interacts with efforts to address health outcomes from a whole school perspective.

## Abbreviations

ALGEE: Assess for risk of suicide or other harm, Listen non-judgementally, Give reassurance and information, Encourage appropriate professional help, Encourage self-help and other support strategies
FSM: Free School Meal
MHFA: Mental Health First Aid
RCT: Randomised Controlled Trial
WISE: Wellbeing in Secondary Education
